# Fiber Optic Sensors Embedded in Textile-Reinforced Concrete for Smart Structural Health Monitoring: A Review

**DOI:** 10.3390/s21154948

**Published:** 2021-07-21

**Authors:** Lourdes S. M. Alwis, Kort Bremer, Bernhard Roth

**Affiliations:** 1 School of Engineering and the Built Environment, Edinburgh Napier University, 10 Colinton Road, Edinburgh EH10 5DT, UK; l.alwis@napier.ac.uk; 2 Hannover Centre for Optical Technologies, Leibniz University of Hannover, 30167 Hannover, Germany; kort.bremer@hot.uni-hannover.de; 3 Cluster of Excellence PhoenixD (Photonics, Optics, and Engineering—Innovation Across Disciplines), 30167 Hannover, Germany

**Keywords:** textile reinforcement, structural health monitoring, fiber optic sensors, smart sensing, sensors in civil engineering, reinforcement of structures

## Abstract

The last decade has seen rapid developments in the areas of carbon fiber technology, additive manufacturing technology, sensor engineering, i.e., wearables, and new structural reinforcement techniques. These developments, although from different areas, have collectively paved way for concrete structures with non-corrosive reinforcement and in-built sensors. Therefore, the purpose of this effort is to bridge the gap between civil engineering and sensor engineering communities through an overview on the up-to-date technological advances in both sectors, with a special focus on textile reinforced concrete embedded with fiber optic sensors. The introduction section highlights the importance of reducing the carbon footprint resulting from the building industry and how this could be effectively achieved by the use of state-of-the-art reinforcement techniques. Added to these benefits would be the implementations on infrastructure monitoring for the safe operation of structures through their entire lifespan by utilizing sensors, specifically, fiber optic sensors. The paper presents an extensive description on fiber optic sensor engineering that enables the incorporation of sensors into the reinforcement mechanism of a structure at its manufacturing stage, enabling effective monitoring and a wider range of capabilities when compared to conventional means of structural health monitoring. In future, these developments, when combined with artificial intelligence concepts, will lead to distributed sensor networks for smart monitoring applications, particularly enabling such distributed networks to be implemented/embedded at their manufacturing stage.

## 1. Introduction

The Paris Agreement aims to address the threat of climate change by keeping the global temperature rise within the century to be below 2 degrees above pre-industrial levels. Environmental impact resulting from human activity continues to make this task challenging. Due to the building industry, concrete is the second-most utilized material in the world, as it requires a high consumption of raw materials and is responsible for a considerable amount of CO_2_ emissions. The production of cement results in 6.5% of the total CO_2_ emissions, which is approximately three times the amount emitted by global aviation [1,2]. Although the construction industry consumes the majority of all resources worldwide, most buildings have a limited lifespan, i.e., of 40 to 80 years. This would mean that most infrastructure scarcely has lifespan longer than that of an average human being, while consuming more energy and resources. The combination of such a relatively short life span and associated costs is unacceptable and unjustifiable in an economical sense, even before environmental impact can be considered. In addition, in line with the global challenge of environmental protection and mitigation of the effects of climate change, new research is being conducted on the utilization of appropriate engineering that reduces CO_2_ emissions incurred by the building industry, while maintaining the safety and durability of building infrastructure. To do so, novel means of concrete reinforcement techniques are researched upon, with low carbon footprint, high reliability in their long-term use and safety.

In conventionally built structures, reinforcement consists of deformed steel bars, i.e., steel reinforced concrete (SRC), with a yield strength of around 450–500 MPa, and is by far the most used combination [3] due to its ductility, low cost, robustness and ease of placement. The ribs/indentations of the deformed bars enable adequate bonding with concrete to transmit the force of the bars to the concrete. This is further facilitated by the fact that concrete and steel reinforcement have a similar coefficient of thermal expansion [3]. The issue with the conventional steel reinforcement, however, is the fact that it is environmentally damaging and corrosive. What is more, steel reinforcement requires a concrete coating to be sufficiently protected against corrosion, thus more concrete needs to be produced for the protection of the reinforcement. A considerable amount of the deterioration of reinforced concrete structures is due to the corrosion of the steel-reinforced bars [4]. Thus, even with a thicker layer of concrete, corrosion is inevitable.

There has been interest in developing fiber-reinforced composites for civil infrastructure in as far back as the mid-1990s, due to their high performance to weight as well as their inherent corrosion and fatigue resistance [5]. Even so, steel-reinforced concrete has been continuously utilized and a competitive alternate, in practice, has not been available. This may also have been owing to the cautious nature of the civil/structural engineering sector who must fully address all risk factors in new technologies, even when effective latest developments are available, albeit within laboratory settings. Therefore, even when advanced technology is present at the laboratory level or indeed through nationally/internationally led research, these developments take a considerable time to reach the industry until they are practically tested and thoroughly evaluated in terms of their feasibility, safety and long-term use.

The last decade, however, has seen various alternative suggestions to SRC and their evaluations on practical implementations which address the issues present in conventional steel reinforcement. With the introduction of non-metallic reinforcement methods, i.e., textile reinforced concrete (TRC), for example, the use of steel reinforcement in building construction is becoming a technique of the past. TRC is a composite material consisting of non-metallic reinforcement and concrete, where the grid-like reinforcement consists of impregnated yarns with up to thousands of filaments. TRC can be characterized depending on the material used, i.e., timber, carbon, alkali resistant glass (ARG), aramid, basalt etc., where carbon and ARG are the most common [6]. In addition to the environmental factors, the life-time durability of TRC depends on various other factors, such as the filament material, i.e., carbon, glass, etc., the impregnation material, and the physical dimensions of the yarns, as well as the production process [7].

When adding textile reinforcement, e.g., made by fiber, to a fine-tuned mortar, a flexible cement composite is produced. This composite would have adequate tensile capacity to the extent that steel reinforcement can be omitted. The advantage of such a result is the capability to access the freedom of shape that is inherent to fluid fresh concrete [8]. The high pH requirement of concrete, in the case of steel reinforcement, is for the protection of the reinforcement bar, i.e., steel, and therefore, if the reinforcement does not have a corrosion possibility, the pH level of concrete would no longer matter. Since glass/polymer does not tend to corrode, there is no requirement for excessive concrete coverage. Therefore, the thickness of structural elements of non-metallic reinforcement can be kept limited to a few centimeters, enabling thin and elegant structures [9] that provide comparable strength while utilizing fewer resources, thus reducing the overall CO_2_ emissions.

As discussed afore, the concepts of non-metallic reinforcement, in particular fiber-based reinforcement, are initiated by the inherent characteristics they possess, which provides a number of advantages over conventional SRC, i.e., conservation of resources, light weight, longer lifespan, thermal and electrical properties, ease of molding despite high strength, flexibility of fabrication, and immunity to risk of corrosion and thus minimum requirements of maintenance. Further, fiber-based reinforcement can be constructed in thin layers with high tensile strength, saving a considerable amount of concrete, i.e., reducing its carbon footprint, while delivering aesthetically flexible construction, which would allow a wider variety for architectural designs. It is predicted that a considerable amount of the concrete volume could be saved this way [10]. Due to the thermal and electrical conductivity of carbon, sensors can be directly installed in the structure, which makes it suitable for intelligent building construction [11,12,13].

However, while offering many advantages, the relatively thinner concrete also poses new challenges, i.e., the current manufacturing technologies might not possess adequate production capabilities to produce these types of reinforcement in bulk and within a short span of time, there might be limited established methods available to ensure successful installations and integration of the increasingly thinner concrete, and the curing methods may require improvements in order to achieve thin concrete layers. Thus, effective and efficient process chains needs to be in place prior to the wider uptake of fiber-reinforced concrete composites by the industry. In addition to these, considerations have to be made on the textile nature of the reinforcement. Grid dimensions have to be carefully chosen to avoid aggregates getting stuck in them [14]. There is also the possibility of abrupt de-bonding of the fabric from the concrete, and thus the bonding characteristics need comprehensive investigation.

The discussion presented thus far is focused on the reinforcement of concrete. One vital element when it comes to reinforcement of civil infrastructure, the majority of which is built with concrete, is termed Structural Health Monitoring (SHM). SHM involves the diagnosis of the “state” of the constituent materials, of the different parts, and of the full assembly of these parts constituting the whole structure, in all stages during its service life [15]. The inherent use of large infrastructure made of concrete, i.e., buildings, bridges, pipelines used for utilities, etc., involves not only the continuous exposure to harsh environmental conditions, but also the bearing of high loads throughout its existence. Any structural damage resulting from whatever cause, may it be natural or man-made, may lead to eventual catastrophic failure, which may cause loss of life. Thus, SHM is needed for continuous damage detection and disaster mitigation. To this end, physical parameters, such as strain, are measured in most reinforced structures to identify structural deformation, i.e., arising from cracks in the concrete and corrosion of the reinforcement [16,17,18]. The suitability of the types of sensors that can be deployed in concrete would depend on factors such as ease of fabrication, the ability to withstand the rigors of being cast into concrete, robustness of the sensor packaging, flexibility of fabrication in a range of geometries and durability against the highly alkaline nature of concrete. Although conventional sensors, i.e., mostly electronic-based, such as strain gauges and accelerometers, have been traditionally employed in SHM systems, these sensors are prone to electromagnetic interference, are tedious to embed, i.e., pools of wires, and could only provide localized, i.e., point, measurements. This would mean that to pick up any indication of a damage, a sensor needs to be present at the location of damage. Thus, with such monitoring systems, there is a considerable likelihood of not obtaining data pertaining to the actual status of the structure.

Fiber Optic Sensors (FOSs), on the other hand, have considerable potential for this purpose and have additional advantages, such as, immunity to electromagnetic interference and their light weight [19]. This is an important aspect for some civil infrastructure, i.e., railways where the lines are electrified [20]. Thus, there has been in depth research on the feasibility and deployment of FOSs for SHM purposes [21,22,23]. The characteristics desired for the ideal FOS for strain measurement in civil structures would include [24]: adequate sensitivity and dynamic range; linear response; sensitivity to the direction of measurand field change; being single-ended, i.e., to minimize the number of leads; insensitivity to thermal fluctuations; non-perturbativity to the structure; immunity to power interruptions; ability to multiplex; ease of mass production, and durability for the entire lifetime of the structure.

The most recent development in FOSs for SHM purposes is the fabrication of textile, i.e., for reinforcement, with sensors already embedded within the textile at the manufacturing stage [25]. Compared to conventional FOSs, which have to be externally bonded to the structure, i.e., such as the case for strain monitoring of bridges [26,27,28], this new technique of sensor-embedded-reinforcement would mean that concrete can be directly poured over the textile and no further implementations would be necessary. Such improvements would not only greatly reduce the labor costs to the service provider, but enhance the monitoring of deformations on a wider area of the structure, i.e., compared to point sensors.

Recent reviews on the subject of new developments in structural reinforcement offer comprehensive information from civil and mechanical engineering perspectives. The available literature on sensors in reinforced concrete had been mainly focused on piezo-electrical-based sensors [29,30,31] and the utilization of the electrical conductivity of carbon fiber [11,12,13]. There is a plethora of literature available on the embedding of FOSs in concrete, especially embedding FOSs on either steel or carbon reinforcement bars. Indeed, currently FOSs are frequently embedded into concrete beams for load tests. These sensors, however, are embedded at a later stage in the lifetime of the structure, i.e., most commonly by attaching the sensors on the surface of the structure. On the other hand, the concept of TRC is relatively new. Embedding FOSs at the manufacturing stage of the structure and directly on a grid-reinforcement inside the arrangement is an upcoming technology, which is not yet widely taken up by the industry. It is our strong belief that the field will increasingly gain interest in the near future as the technology to embed sensor systems into TRC continues to be improved and widely used, although its potential is still far from being fully exploited. This is evident from the significantly limited literature on the topic of FOSs embedded in the latest structural reinforcement techniques, particularly in TRC, at the manufacturing stage. This communication aims to address this gap and provide the state-of-the-art and challenges faced when utilizing FOSs for the SHM of civil structures, particularly on structures made using TRC.

## 2. Textile-Based Concrete Reinforcement

In TRC, multi-axial textile fabrics are used in combination with high-strength fine-grained concrete. Typically, a TRC substrate consists of a matrix with a maximum aggregate grain size between 1 and 2 mm and high-performance continuous multifilament yarns made of ARG, carbon, or polymer [32]. Multi-axial reinforcement fabrics are built from multiple filament yarns, i.e., rovings, which contain several hundred to several thousand individual filaments of roughly 5–25 µm in diameter [32]. These yarns can be placed according to the expected stresses by varying their amount and the filament orientation [33]. The thus fabricated rovings structure provides sufficient dimension of contact with the concrete composite. The fibers would typically be placed in the main stress direction of the composite, which leads to a higher effectiveness in comparison to the use of randomly distributed short fibers [34]. The main advantages of TRC are its high tensile strength and flexible ductile behavior, which enables relatively thin-structured concrete elements, as described in Section 1. TRC has been extensively investigated, particularly by TU Dresden and RWTH Aachen University, for nearly two decades to date [35,36,37], where the potential for its use in the industry is well confirmed [9].

The inherent characteristics of the fiber material used, and the amount and the arrangement would influence the performance of the composite TRC to ensure its safe operation within the expected lifespan. The material should also withstand the alkaline medium while maintaining its inherent properties. In addition, the modulus of elasticity of the fiber and the tenacity and ductility of the fiber should be taken into account when potential candidate materials are considered. For example, the modulus of elasticity of the fiber should be higher than that of the concrete matrix. Failure to meet this requirement could result in the formation of cracks that would have detrimental effects on the stiffness of the concrete/structure. The fiber should also possess adequate bonding capabilities between the reinforcement itself and the concrete so that effective transfer of the measurand data could be achieved. Other practical considerations would involve cost effectiveness and ease of manufacturing. The use of ARG and carbon for the design and fabrication of textile reinforcements essentially meets these requirements [34].

**Figure 1:**
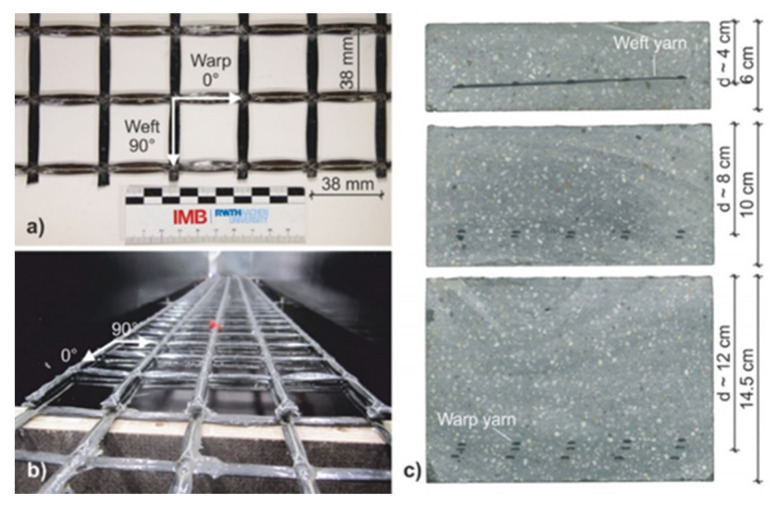
Biaxial textile carbon grid (**a**,**b**), as well as the cross-section of the concrete specimens (**c**) [6].

**Figure 2:**
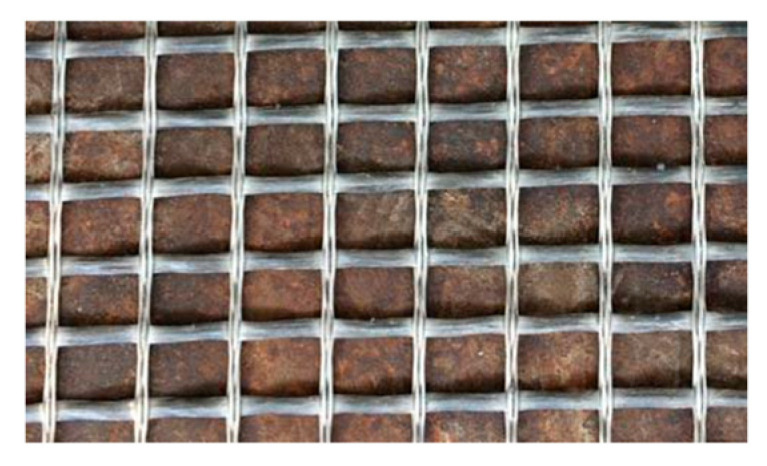
Alkali resistant glass fiber textile [40].

Thus, it is of no surprise that the two most used materials for reinforcement in TRC are ARG and carbon. ARG typically includes 15–20% of zirconium, which improves the durability of the glass in alkaline environments [38]. The number of filaments in one roving follows the filament diameter and the fiber coarseness in g/km (tex, Titer), where, with a diameter of about 27 µm, a 2400 tex roving consists of nearly 1600 filaments, as shown in Figure 1. Given such minute dimensions possessed by the fiber strands, further investigations would be necessary to evaluate whether their physical parameters affect their functionality. To that end, Scheffler et al. [39] provided a detailed analysis on the effects of varying the diameter of ARG fiber, see Figure 2 for an example, and its influence on surface sizing and coating. They achieved an improved durability of the ARG fibers by nano-coatings based on self-crosslinking styrene–butadiene polymer, where the sizing and compatible coating on ARG and carbon fibers are characterized as the basis for increased tensile strength that is also influenced by the filament diameters and the thickness of the sizing/coating layer. Tailored sizing and compatible coatings provide a basis to achieve enhanced mechanical performance for both glass and carbon, while nanotubes in the sizing enhance the fiber tensile strengths additionally.

There has also been some interest on the practical applications of TRC. For example, Cauberg et al. [7] presents work on the production of thin, doubly curved shell elements that can be used as façade panels, self-bearing roofing structures, or permanent formwork elements. It would be challenging to produce and reinforce these with conventional techniques. One main advantage of utilizing glass fiber TRC shells is that the thickness of the concrete will not be restricted by corrosion cover recommendations, i.e., as is for steel-reinforcement. Thus, the combination of TRCs with a flexible fabric formwork enables curved shells, and indeed other shapes, without the conventional limitations on the shape or thickness. Another example of TRC in practice is the work by Pan et al. [41] who presents an experimental investigation on the impact fatigue behavior of the glass fiber polymer mesh-reinforced Engineered Cementitious Composites (ECC), i.e., a cement-based ductile composite with a strain hardening behavior in tension, for runway pavement applications under the design aircraft pressure.

**Figure 3:**
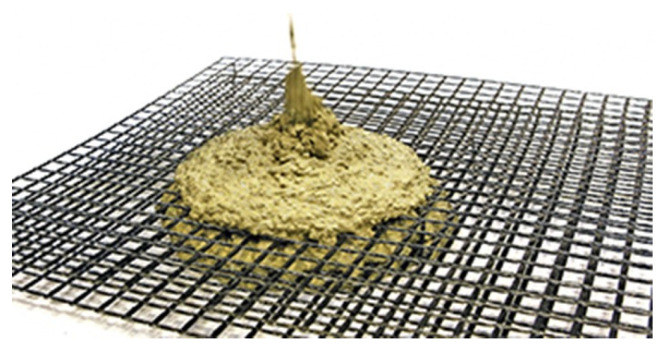
Illustration of the flow process of the fresh concrete through and between the layers of close-knit carbon fabric reinforcement. Reprinted with permission from ref. [44]. Copyright 2017 Beton und Stahlbetonbau.

In terms of TRC based on carbon fiber, the composite material consists of high-performance concrete and carbon. Carbon reinforcement has very low weight, i.e., density is four times lighter than of steel, has a high load-bearing capacity, and is resistant to corrosion, enabling the reinforcement to be positioned close to the surface [42]. Thus, it has the desirable feature of facilitating thinner structural designs. It can be estimated that by the use of carbon concrete composites, material consumption can significantly be reduced while improving durability [42]. Projects such as the Carbon Concrete Composite (C^3^) initiative in Germany [1,2] aim to investigate the possibility of using CRC as an alternative to traditional steel reinforcement in building construction. However, in order to facilitate mass production and wider uptake of C^3^, it is necessary to maximize the process of C^3^ manufacturing, i.e., from carbon fiber manufacture to the production of the C^3^. A recent study by Bohm et al. [43] explores efficient means to manufacture carbon fiber for the purpose of TRC in detail, compare Figure 3 for one of several possible configurations.

Another interesting recent development in the building industry is the 3D printing of concrete and specifically those with textile reinforcement in place. Formwork, i.e., used for the casting of concrete, constitutes approximately 60% of the total cost of concrete construction [45]. As such, it represents a significant source of waste worldwide. With latest advances in robotics, automation and building-scale additive manufacturing processes, the construction industry is seeing a rapid uptake of the state-of-the-art for improved efficiency, flexibility and safety [46]. Thus, 3D-printed concrete has gained significant interest in both the academic and commercial arenas. However, as well as the technology itself, much attention is focused on the equipment and material with which 3D printing of concrete can be made efficient, economical and practical [47].

**Figure 4:**
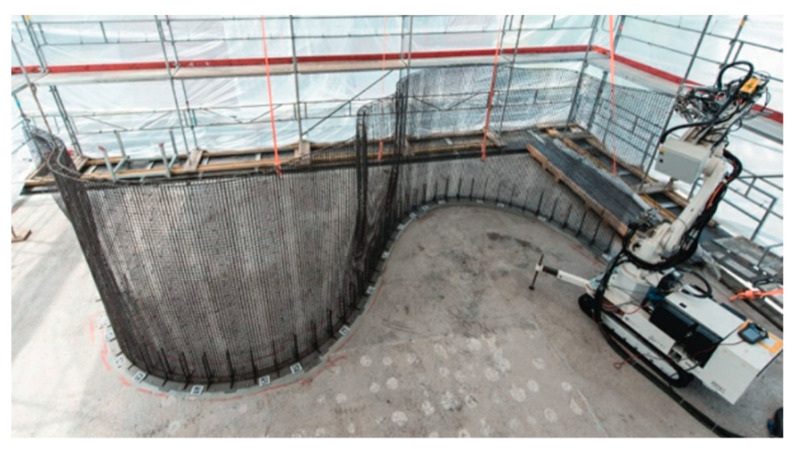
Production of 14 m long mesh for a double curved load-bearing wall for the DFAB HOUSE, Switzerland. Reprinted with permission from ref. [49]. Copyright 2018 Cement and Concrete Research.

**Figure 5:**
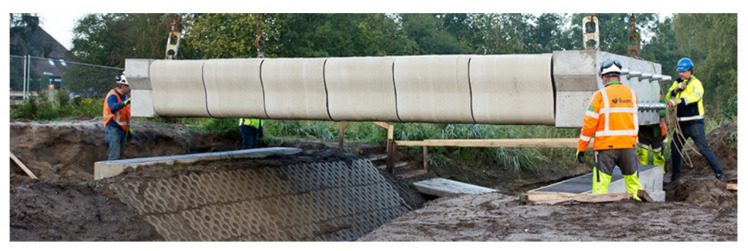
The 3D concrete printed pedestrian and bicycle bridge in Gemert, the Netherlands, is hoisted into position. Reprinted with permission from ref. [3]. Copyright 2018 Cement and Concrete Research.

Asprone et al. [3] introduces the term “Smart Dynamic Casting” (SDC) as a robotic prefabrication technique for concrete structures that are non-standard. The adaptable rheology of fresh concrete is utilized by pouring it onto a moving formwork. At the bottom of the formwork, concrete is in a hydrated state but has adequate strength to be self-sustaining. Finally, the hydration process is digitally controlled by an automatic sensor system [48]. Thus, SDC enables digital fabrication of complex column structures in a continuous casting process. The main challenge in constructing these structures is the need to deform the steel bars according to the complex geometry of the vertical element. However, this challenge is addressed using robotic reinforcement techniques, i.e., mesh mold technology. In [3], the reinforcement integrated in the SDC technique is fabricated before the casting of concrete. This was achieved by the utilization of numerically controlled bending processes in 3D. The advantage of such a scheme is the fact that is allows the utilization of standard and inexpensive deformed steel bars, even for complex structures. One such example is the production of variable-cross-Section 3 m tall mullions for the DFAB HOUSE [49] in the NEST building at Empa in Dübendorf, Switzerland, see Figure 4. Another example is shown in Figure 5. 

Although steel reinforcement is used for SDC at present, the uptake of TRC for the purpose would widen the potential for SDC technology in many aspects. For example, the main challenge of deforming the steel bars according to the geometry of the structure can be easily achieved using TRC. The corrosion possibility of the steel reinforcement can also be fully eliminated using TRC, which would also minimize the amount of concrete that needs to be used, i.e., a thick layer of concrete is needed in order to protect the steel bar. This would enable 3D-printed structures utilizing TRC, having a low carbon footprint and being elegant in their design. However, TRC may lack the tensile strength and ductility that is needed for complex geometric shapes and therefore might have limitations on its uptake. Thus, this is an area of research that is still in its infancy and requires extensive exploration prior to its use in the industry.

The discussion presented thus far showcases the progression of concrete reinforcement from conventional steel reinforcement to the most recent developments in the building industry, i.e., 3D printing of concrete structure. As highlighted in the introduction section, and having had a brief description on state-of-the-art on textile reinforcement, the subsequent section of this communication focuses on the utilization of FOSs for the SHM of civil infrastructure, particularly those equipped with TRC.

## 3. Fiber Optic Sensors (FOSs) for the SHM of Concrete Structures

### 3.1. Optical Fiber Technology and Sensors

The development of optical fiber saw a rapid increase in the 1990s for telecommunications purposes. Indeed, most of the optical sources, detectors, and related consumables that are still in use, i.e., connectors, couplers, etc., operate at the wavelengths that were initially focused on telecommunication windows, i.e., 1550 nm and 1300 nm. In recent years, fiber optics has also drawn considerable attention in the sensors field owing to its many advantages over the conventional electrical-based sensors, such as immunity to electromagnetic interference, multiplexing capability, high temperature capability, chemical inertness, robustness, and light weight. A FOS system consists of a transducer device, i.e., the sensing element or mechanism, a communication channel which carries the measurand data, and a subsystem that provides energy and detects/processes and conditions the received signal. Since several sensors can be embedded along a single fiber, creating a distributed sensors network, the detection of local damage such as strain, cracks, and corrosion, becomes a possibility. The conventional electrical sensors must have two wires for each sensor to form an electrical loop. In comparison, the multiplexing capability of the optical fiber network is far easier to implement and install. FOSs can be used either as point sensors or in a distributed sensor configuration, making the design of the sensor or the sensor network more flexible and application specific. Thus, depending on the transduction mechanisms used, a variety of physical and chemical sensors can be developed, which have potential applications in a wide range of industries.

**Figure 6:**
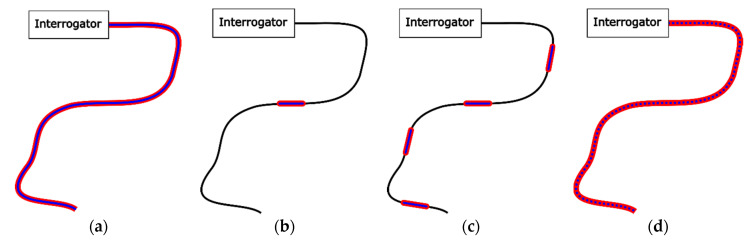
Graphical illustration of different FOS concepts: integrated (**a**), point (**b**), quasi-distributed (**c**), and distributed (**d**). The black line illustrates the fiber optic link. The red and blue lines represent the sensing length and measurement points respectively.

The use of FOSs for the SHM of concrete was first suggested by Mendez et al. (1990) [50]. Since the first development from Mendez et al., many different FOSs have been conceived for SHM of concrete through the measurement of cracks [51], strains [52,53] as well as Relative Humidity (RH) [54] and pH [55,56], for instance. In general, the FOS concepts can be categorized into (i) integrated, (ii) point, (iii) quasi-distributed and (iv) distributed sensing, depending on the spatial distribution and sensing mechanism [57]. The different categories of FOSs and their corresponding sensing mechanisms are summarized in Figure 6, followed by an explanation on their operating mechanisms and their utilizations in exemplary applications.

### 3.2. Integrated FOSs

**Figure 7:**
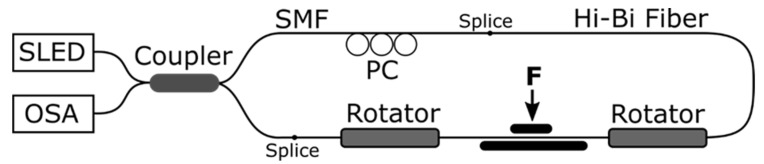
Set-up of a fiber optic Sagnac interferometer that has been applied for the corrosion monitoring of steel reinforcement bars [60].

In case of integrated FOSs, the measurement is integrated along the whole length of the optical fiber that is exposed to the measurand, i.e., when the FOS element is exposed to spatially different stimulus, only one final total measurement value is obtained. Typical examples of integrated FOSs are intrinsic Sagnac and Mach-Zehnder interferometric sensors. The term ***intrinsic*** means that the throughput properties are modulated by an impacting environmental signal where the interaction (sensing) between the light and the target measurand takes place within/with the fiber and thus the optical fiber itself would be the sensor. For instance, a fiber optic Sagnac Interferometer (SI) usually consists of a polarized broadband light source, a 3 dB fused fiber coupler, a Polarization Controller (PC), an Optical Spectrum Analyzer (OSA) and a fiber optic sensing element based on High Birefringent (HB) Polarization Maintaining (PM) fibers. Light from the optical source is split into two counter-propagating beams using the fused fiber coupler. The two counter-propagating beams propagate through the optical fiber element and recombine at the fused fiber coupler, where the corresponding interference pattern is recorded using the OSA. The phase of the interference pattern depends on the birefringence of the applied optical fiber. The PC is used to control the polarization of the light source. Fiber optic SIs have been applied to determine parameters such as strain [58] or pressure [59], for instance. Moreover, when applying low-temperature sensitive HB PM Photonic Crystal Fibers (PCFs), the cross-sensitivity to temperature can be compensated [58]. In terms of SHM of concrete structures, fiber optic SIs have been successfully used to measure the corrosion of steel bars, as shown in Figure 7 [60].

**Figure 8:**
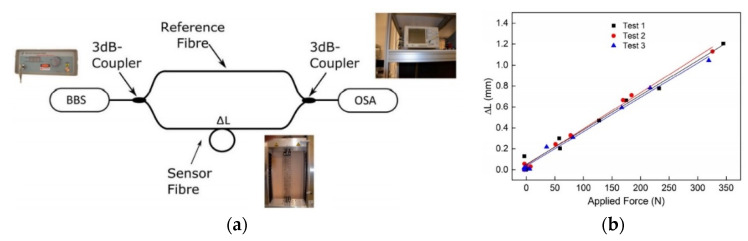
Fiber optic Mach-Zehnder set-up to measure and characterize the strain transfer between a textile-based carbon reinforcement structure and the optical glass fiber: (**a**) Experimental set-up and (**b**) obtained sensor response [51].

In contrast, a fiber-optic Mach-Zehnder Interferometer (MZI) can be built by using two 3 dB couplers that split the light from an optical source to single-mode fiber optic reference/sensor arms as well as recombine it to create an inference pattern at the detector. Any induced length change of the measurement arm due to thermal expansion, force, or strain causes a phase difference between the light traveling in both fiber arms, which in turn results in an interference pattern change. In general, the integrated interferometric techniques have the benefit of allowing a relatively accurate and cost-efficient approach to the characterization of FOSs that are embedded into concrete structures in SHM applications. A fiber optic Mach-Zehnder set-up that was built to characterize the strain transfer between a textile-based carbon reinforcement structure and an optical glass fiber is shown in Figure 8.

### 3.3. Point-Based FOSs

Point-based FOSs are used when the measurement is only obtained at a discrete point along the structure to be monitored. This concept can be designed to operate in transmission or reflection mode, depending on the requirement/application. In addition, it has the advantage that, depending on the principle of operation, point-based FOSs can be designed to be relatively low-cost, robust and compact.

#### 3.3.1. Grating-Based FOSs

Grating-based FOSs consist of a periodic perturbation of the refractive index of the optical fiber core [61]. This modulation of the optical fiber core can be formed by different laser exposure techniques [62]. In general, depending on the coupling properties, the mechanism of the grating-based FOSs can be categorized into co- and counter-propagating coupling [61]. In case of co-propagating coupling, light is coupled between modes propagating in the same direction within the optical fiber. Since the grating periods that are required to compensate for the propagation constant mismatch between the modes to be coupled are in the order of several hundred micrometers, this type of grating structure is called Long-Period Grating (LPGs). In contrast, for counter-propagating coupling, light is coupled between two counter-propagating modes within the optical fiber and the corresponding fiber element is called a Fiber Bragg Grating (FBG).

**Figure 9:**
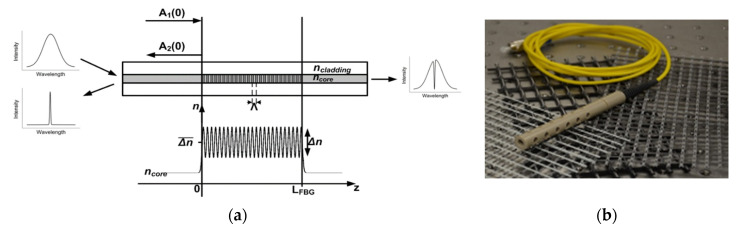
(**a**) Principle of operation of FBGs: periodic modulation of the refractive index of the fiber core which causes that only the Bragg wavelength is reflected and all other wavelengths to propagate through to the end of the optical fiber. (**b**) Example of a packaged FBG sensor that has been applied for the measurement of relative humidity. Reprinted with permission from ref. [68]. Copyright 2016 Procedia Technology.

In terms of SHM applications, FBG sensors are the most mature grating-based sensors and have been applied to measure parameters such as strain [63,64], temperature [65], and RH [21,54], among others. When light is coupled into a FBG, only light of a particular wavelength is reflected (the so-called Bragg wavelength), while the rest propagates through to the end of the fiber, as shown in Figure 9. The Bragg wavelength is defined by the effective refractive index of the modes to be coupled as well as the grating period. Both parameters depend on external perturbations, temperature, and strain and therefore by monitoring the shift of the reflected Bragg wavelength, the change of the external perturbation, i.e., temperature or strain, can be monitored. Moreover, since a FBG is sensitive to two measurands, i.e., temperature and strain, two FBGs with different material properties [66] or packaged on an asymmetric elastic substrate [67], can be applied to distinguish between the two measurands and thus to compensate for this inherent cross-sensitivity.

#### 3.3.2. Point-Based Interferometric FOSs

**Figure 10:**
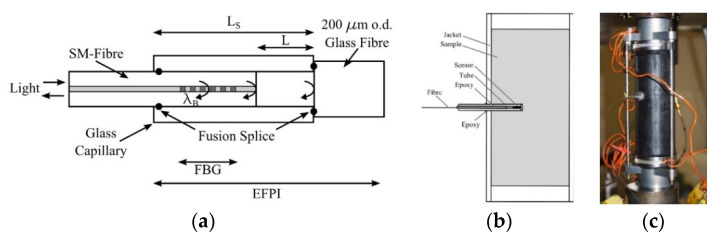
Schematic of an EFPI FOS that was designed for the measurement of pressure [71]: a FBG FOS was integrated inside the EFPI in order to compensate for temperature cross-sensitivity (**a**). The developed FOS was applied to measure pressure and temperature inside rock samples (**b**,**c**) and thus to characterize their. Reprinted with permission from Ref. [71]. Copyright 2012 International Journal of Rock Mechanics and Mining Sciences.

Compared to integrated interferometric FOSs, point-based interferometric FOSs consist of a light modulator element as well as input and output fibers, in order to carry light to and from the light modulator. Since the light modulator element converts physical changes to phase differences between two interference light waves, the light modulator acts as a sensing element. Therefore, the optical fiber only represents the light transmission medium and external stimulus can be monitored by discrete sensing elements along the optical fiber link. This is termed as an ***extrinsic*** sensor setup. With respect to SHM, the most common point-based interferometric FOS is the so called Extrinsic Fabry-Perot Interferometer (EFPI) [69]. Compared to intrinsic sensor elements, in the case of extrinsic sensors, the light propagating inside the optical fiber is modulated by an external/extrinsic optical element. EFPI FOSs can be realized by two optical fibers that are separated by a gap of usually several tens to hundred micrometers and are aligned and mounted using a glass capillary. The optical end-faces of the optical fibers results in a low-finesse interferometer where any length change of the gap between the two fibers due to thermal expansion, strain, or pressure is converted into a phase difference of the interferometric signal. One advantage of EFPI FOSs is their capability to operate even at very high temperatures [70]. Furthermore, the fully packaged EFPI FOS can be designed to be compact with a length of only a few millimeters and hence can be embedded into structural components without impacting their properties. An EFPI FOS that was used to measure pressure and temperature inside rock samples, is shown in Figure 10.

#### 3.3.3. Micro-Bend FOSs

**Figure 11:**
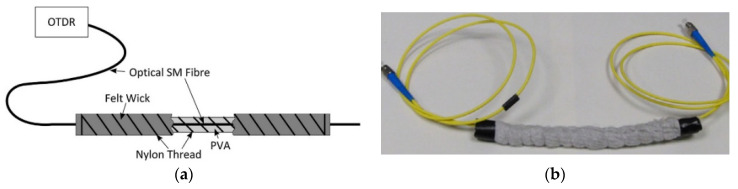
A fiber optic leakage sensor that is based on the micro-bend principle: An optical glass fiber and a PVA rod were bonded together using a nylon thread and covered by a felt wick. When the sensor is exposed to water, the PVA swells and presses the optical glass fiber against the nylon thread and thus induces attenuation of light. (**a**) Schematic of the FOS; Reprinted with permission from Ref. [54]. Copyright 2014 Sensors and Actuators A: Physical. (**b**) Picture of the manufactured FOS; Reprinted with permission from Refs. [54,68]. Copyright 2014 Sensors and Actuators A: Physical and Copyright 2016 Procedia Technology.

In case of micro-bend FOSs, optical losses are induced by the measurand that modulates the amplitude of the light propagating inside the optical fiber and, thus, modulates the power loss of the optical fiber link. In order to modulate the amplitude of the light propagating inside the optical fiber, a transducer mechanism, which can be based on two plates with saw-shaped edges [72] or spiral wires wrapped around the optical fiber [54,68,73], is applied. In both cases, the bending of the optical fiber causes light coupling from the core mode to the cladding modes, which are highly attenuated by the primary coating of the fiber and, thus, induces light attenuation as a direct result of the bend experienced by the fiber. A schematic, as well as a picture of a fiber optic leakage sensor based on the spire wire technique, is illustrated in Figure 11 [54]. The fiber optic leakage sensor was developed for the SHM of sewerage pipes.

#### 3.3.4. Other Point-Based FOSs

Chemical FOSs have also been developed for SHM. In case of the SHM of concrete structure, chemical parameters such as pH, carbonation, or corrosion would be of interest [57]. In order to measure these parameters, a direct or indirect detection technique can be applied. The direct detection mechanisms would work only when the measurand is able to directly modulate the optical light by its spectroscopic fingerprint, for instance. In case of indirect measurement techniques, an intermediate material is applied, whose mechanical or optical properties depend on the measurand. Examples of intermediate materials are polymer, hydrogels, and dye indicators. For instance, polyimide (PI) has been applied for RH measurements [21,54,68]. PI has the property of swelling when exposed to humidity and thus would induce a strain, which can then be measured using a FBG configuration. Hydrogel polymers have the inherent characteristic of varying their optical absorption, refractive index, and volume when exposed to moisture [57], for instance. In addition, in order to measure pH, dye indicators can be applied and depending on the pH concentration the detectable fluorescence would change [74]. To monitor the change of the optical properties of the intermediate material, different fiber optic sensing schemes can be applied, employing, for example, FBGs, LPGs, tapered optical fibers, or simply optical fiber end-faces.

### 3.4. Quasi-Distributed FOSs

**Figure 12:**
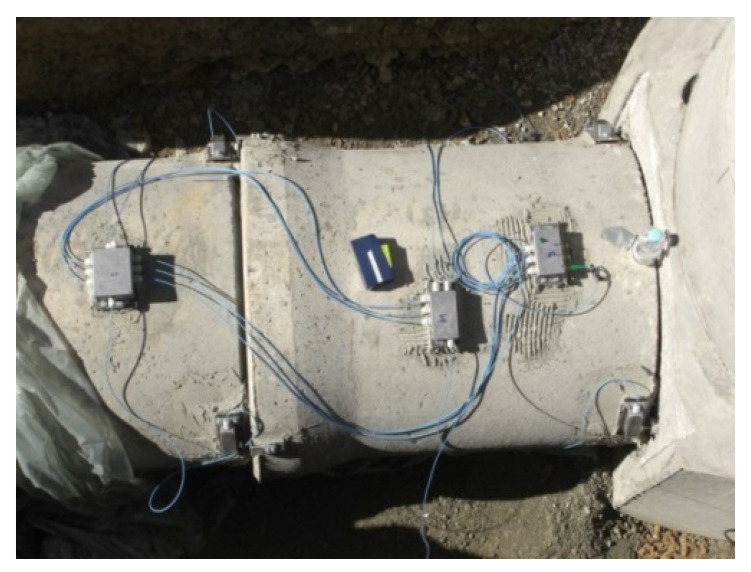
Example of a quasi-distributed FBG sensor network that was developed for the SHM of sewerage tunnels. Reprinted with permission from Ref. [68]. Copyright 2016 Procedia Technology.

Quasi-distributed FOSs consist of a network of multiplexed point-based FOSs and allow the continuous monitoring of large structures, see Figure 12. Furthermore, since many point-based FOSs share the same interrogation unit, quasi-distributed FOSs networks can also become more economically efficient. The multiplexing techniques for quasi-distributed FOSs are adopted from optical fiber communications sector and are mainly based on Wavelength Division Multiplexing (WDM) [75], Time Division Multiplexing (TDM) [76], frequency domain multiplexing (FDM) [77], Code Division Multiplexing (CDM) [78] or a combination of these techniques. For instance, by combining WDM and CDM a sensor network consisting of 2000 FBG sensors could be interrogated [79]. In terms of SHM applications, the condition of a sewerage tunnel could be monitored [68] or the eigen-modes of a marine propeller could be studied using several FBG sensors that are multiplexed using WDM [80].

### 3.5. Distributed FOSs

**Figure 13:**
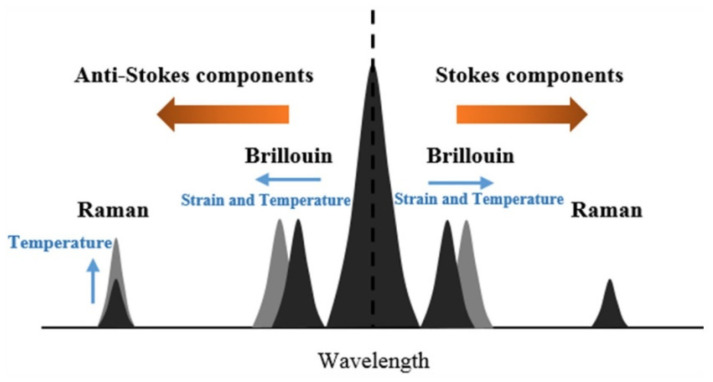
Illustration of the different frequency components in a signal due to Rayleigh, Brillouin, and Raman scattering. Reprinted with permission from Ref. [85]. Copyright 2016 Measurement Science and Technology.

For the SHM of large infrastructure such as railway tracks [81], pipelines [82], dikes [83], tunnels [84], and bridges [85], distributed fiber optic sensing approaches are the best candidates since they allow a continuous measurement profile over the entire length of the optical fiber. Therefore, in this case, the optical fiber acts as both the transmission and sensing medium. Depending on the physical effects of the operating principle, distributed fiber optic sensors can be categorized into (i) Rayleigh scattering, (ii) Brillouin scattering, and (iii) Raman scattering, compare Figure 13.

#### 3.5.1. Rayleigth Scattering-Based Distributed FOSs

The elastic scattering of light by particles much smaller than the light wavelength is known as Rayleigh scattering and forms the basis for sensing techniques such as Optical Time Domain Reflectometry (OTDR) and Optical Frequency Domain Reflectometry (OFDR). In the case of standard OTDRs, an optical light pulse, from a light source which has a coherence length that is shorter than the pulse length, is coupled into an optical fiber and the backscattered light due to the Rayleigh scattering is recorded using a photodetector. When the received light is recorded with a time stamp, the attenuation profile of an optical fiber link can be evaluated, through which the location of splices and connectors as well as breakages of the optical fiber link, can be determined. For instance, the standard OTDR technique in combination with a fiber optic leakage sensor has been proposed for distributed SHM of sewerage tunnels [54]. In the event of a leak in the sewerage tunnel, the leakage sensor introduces an attenuation in the optical fiber link and, by using the OTDR, the location of the attenuation, and thus the leak, can be detected. Another technique used for the purpose is Coherent Optical Time Domain Reflectometry (COTDR). In this case, the coherent length of the applied light source is longer compared to the pulse length and, thus, the optical fiber acts as a distributed interferometer with a gauge length almost equal to the pulse length. Therefore, external stimulus acting on the optical fiber causes a variation in the amplitude and phase of the backscattered light, which can be detected in the backscattered light profile of the COTDR. This type of system has high sensitivity to strain and temperature changes and is applied, among others, for Distributed Acoustic Sensing (DAS). In terms of SHM, DAS is applied for large infrastructures such as pipelines [86]. In contrast, the OFDR is a hybrid technique based on Rayleigh backscattering and swept wavelength interferometry [87]. Here, a swept laser source and an interferometer are applied to measure Rayleigh backscattering as a function of the optical fiber length. External stimulus, such as strain and temperature, causes a shift in the measured Rayleigh backscattered length profile of the optical fiber and, thus, a distributed strain and temperature profile can be obtained.

#### 3.5.2. Brillouin Scattering Based Distributed FOSs

Brillouin scattering is a nonlinear phenomenon caused by the interaction of acoustic waves and the monochromatic light propagating inside the optical fiber. The acoustic waves responsible for the interaction can be created by spontaneous thermal motions of particles and their propagation causes a periodic modulation of the refractive index of the optical fiber. This in turn leads to light scattering that has a frequency shift compared to the incident light (Brillouin frequency shift). Furthermore, the shift of the Brillouin frequency depends on the properties of the optical fiber, i.e., stress and temperature. Different distributed FOSs based on Brillouin scattering have been reported, such as Brillouin Optical Time Domain Reflectometer (BOTDR) [88] and Brillouin Optical Time Domain Analysis (BOTDA) [89]. In case of BOTDR, a light pulse is coupled into an optical fiber, which acts as both the sensing and the light guiding medium, and is partially scattered back due to Brillouin scattering when propagating along the optical fiber. Since the Brillouin frequencies of the partially scattered light depend on the local temperature and strain profile of the optical fiber, the BOTDR is capable of measuring both parameters by mapping the measured Brillouin frequency and its time of arrival. In comparison, the BOTDA technique uses stimulated Brillouin scattering that occurs when the frequency difference between a pulsed pump laser and a counter-propagating continuous wave probe laser is equal to the Brillouin frequency of the optical fiber and causes an amplification of the probe laser. Therefore, when scanning the probe laser frequency and measuring the increase in the continuous light as a function of time, the Brillouin spectrum of the optical fiber can be measured as a function of space, resulting from the spatial frequency shift due to strain and/or temperature changes.

#### 3.5.3. Raman Scattering Based Distributed FOSs

When light propagates inside an optical glass fiber, part of the light is backscattered due to molecular oscillations at a wavelength shift relative to the incident light wavelength. The latter effect is the so-called Raman scattering, which is another nonlinear phenomenon. Raman scattering consists of a Stokes and an anti-Stokes band. The intensities of both bands depend on the temperature, with the anti-Stokes band showing a higher temperature sensitivity than the Stokes band. Therefore, the temperature at any location along the optical fiber can be determined from the ratio of the intensities of the anti-Stokes and Stokes bands. When using the Raman Optical Time Domain Reflectometry (ROTDR) [90], the temperature profile along the optical fiber can be measured as a function of location and time.

## 4. Functionalizing Textile-Based Reinforcement Structure with FOSs

On the basis of our discussion in Section 1, FOSs have many advantages compared to conventional electrical-based sensors, especially in the civil engineering sector. However, although FOSs are ideally suited for SHM of reinforced concrete, the logistical challenges during their installation and construction must be addressed. For example, the fiber leads are vulnerable to breakage at the point of exit from the concrete. This would demand appropriate protection, not only due to the exit point, but also due to the harsh environment the sensors are most likely be exposed to, during installation as well as throughout its lifespan. Therefore, the direct integration of FOSs into concrete forms a new field of research which requires considerations on the durability of the said integration and thus its practicality in the long-term use. For example, it is of vital importance to evaluate whether the physical construction of the optical glass fiber is able to withstand its host environment, i.e., concrete.

The combination of FOSs and geogrids for the strengthening of geotechnical structures e.g., [91,92,93] or FOSs and reinforcing composite patches based on either fiber reinforced plastics (FRPs) [94] or Fiber Reinforced Cementitious Matrix (FRCM) [95], for the rehabilitation of concrete structures, have been already explored in the literature. However, the integration of optical fiber sensors in TRC is still in the early stages of development, and thus, there is no sufficient literature available to compare their performance, structurally or in terms of efficiency and economy. Nevertheless, given the trend with new reinforcement techniques, combined with potential digital fabrication of concrete, it is possible to discuss their potential.

### 4.1. Fabrication Techniques

**Figure 14:**
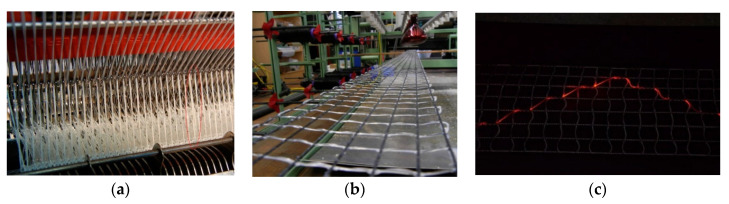
Fabrication of TNS: warp knitting machine (**a**), fabricated biaxial grids consisting of alkaline-resistant glass multifilament and a polypropylene multifilament thread (**b**), and the TNS with integrated optical glass fiber (**c**). Reprinted with permission from Ref. [51]. Copyright 2016 Procedia Technology.

The incorporation of optical fibers in concrete can either be performed manually afterwards, or automatically during the fabrication of the reinforcement structure. However, in terms of the mass production of large reinforcement structures, the latter incorporation technique is more economical. A considerable amount of work on FOSs-integrated TRC has been conducted by the Saxon Textile Research Institute (STFI) in Chemnitz, Germany, who has developed different techniques for the automatic incorporation of optical fibers into textile-based reinforcement structures. For the integration of optical fibers into Textile Net Structures (TNS), STFI has developed an appropriate reel-to-reel knitting technique [51,68]. TNSs are biaxial grids consisting of alkaline-resistant glass multifilament that are arranged in 0° and 90° directions and a polypropylene multifilament thread. They are fabricated using a warp knitting machine and stabilized afterwards by applying a copolymer coating. For the integration of optical fibers, the STFI fabricated a TNS with a spacing of 20 mm and a 2400 tex alkaline-resistant glass multifilament as well as a 44 tex multifilament thread. The optical glass fiber was integrated during the knitting process of the TNS. The stitching technique not only enables adequate bonding between the optical fiber sensor and the TNS, but also results in low light bend losses into the optical fiber. The warp knitting machine that was applied to fabricate the TNS, i.e., with embedded FOSs, is shown in Figure 14 [51].

**Figure 15:**
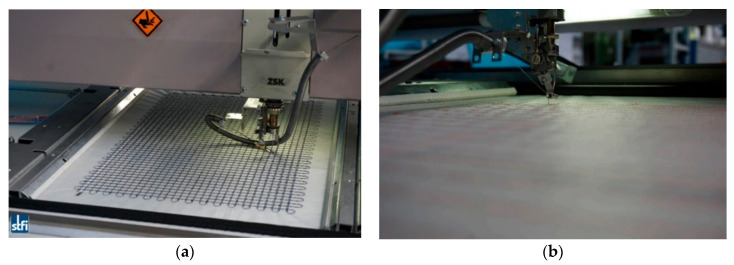
Modified embroidery machine (**a**) that was developed for simultaneous processing of the carbon fiber filaments and optical fibers on PVA nonwoven substrate (**b**) [51].

For the fabrication of functionalized carbon structures (FCS), i.e., textile-based reinforcement structures consisting of carbon filaments and functionalized with optical glass fibers, an appropriate embroidery technique was also developed at STFI [51]. In this case, the optical fibers were “woven” simultaneously while manufacturing the textile-based reinforcement structure based on carbon filaments to produce grid-like elements. The fabrication technique developed allows the fabrication of tailored structures in that several layers of carbon filaments in different shapes can be manufactured and hence the reinforcement structures can be optimized for the targeted application. The modified embroidery machine is illustrated in Figure 15. This is particularly developed for simultaneous processing of carbon and optical fibers [51]. The interwoven carbon/optical-fiber strands were obtained by embroidering the optical fiber and carbon filaments on a Polyvinyl Alcohol (PVA) nonwoven substrate. In order to obtain the grid-like structure, multiple layers of carbon fibers were then integrated on the PVA substrate, which was removed by dissolving the PVA in hot water (in the range of 50 °C) upon completion of the fabrication process. The fabricated functionalized grid reinforcement structure is shown in Figure 15. As can be seen, the grid was embroidered on the PVA substrate as the initial step.

### 4.2. Resistance against Highly Alkaline Concrete Environment

The water in the pores of Portland cement is alkaline with a pH in the range between 12.5 and 13.5 [96]. Therefore, when functionalized TRCs are embedded into concrete, the highly alkaline environment might degrade the mechanical properties of both the optical glass fiber and the textile filaments as well as the bond between the two components, thus limiting the life-span and operation of the combined sensing and reinforcement structure. Therefore, the impact of highly alkaline concrete environments on the functionalized textile-based carbon reinforcement structure was investigated in [96]. In this study, the resistance of the functionalized textile-based reinforcement structure against the highly alkaline environments was explored depending on the coating of the optical glass fiber (acrylate, polyimide and carbon) and the tex-number of the textile filament (400, 800 and 1600 tex) of 300 mm long one-dimensional structures that have been fabricated according to the technique described in Section 4.1 [96]. Moreover, in order to simulate the highly alkaline concrete environment, a 5% NaOH solution (pH 14) was used in this study and the samples were exposed to this solution over a period of three months. From the investigation in [96], the authors deduced that optical glass fibers with carbon coating showed the best resistance against highly alkaline pore water [96]. Moreover, since PVA is used for the fabrication of the functionalized textile-based reinforcement structures (which is relatively inert against chemicals), the functionalized reinforcement structures are relatively stable against alkaline pore water attack in general [96]. The best resistance was obtained for textile-based reinforcement structures with higher tex numbers [96].

### 4.3. Different Fiber Optic Configurations

**Figure 16:**
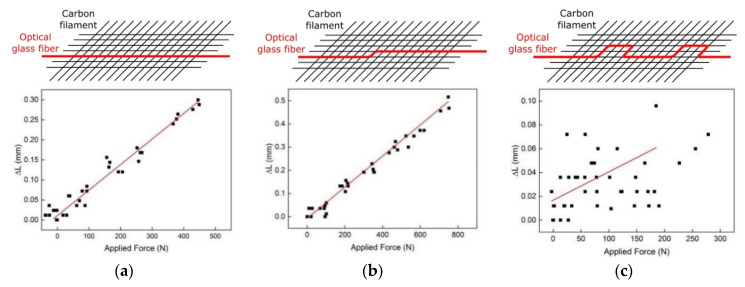
Responses of the functionalized textile-based reinforcement structure to applied force by different fiber optic sensor configurations: straight (**a**), offset (**b**) and meander (**c**) [96].

In order to investigate whether the shape of the optical fiber inside the TRC has any impact on the sensor response, three different integration configurations of the optical fiber were explored in [96]. All three different optical fiber configurations are shown in Figure 16. The first configuration is a straight optical fiber whereas in the second and third configurations the optical fiber was integrated with an offset or a meander, respectively. The latter two cases were chosen in order to investigate spatial variations and whether such variations cause an optimized sensor response. The functionalized TRC structures were fabricated using 1600 tex carbon filaments and acrylate coated optical SM glass fibers (Corning CC) and the final size of each structure was 500 × 110 mm^2^ with a grid size of 10 mm × 10 mm. The characterization of the optical fiber sensor response was analyzed using a fiber optic Mach-Zehnder interferometer approach and the obtained results are shown in Figure 16 [96].

For the straight (first case) and offset (second case) optical fiber configurations, linear responses to applied force of 6.4 × 10^−4^ mm/N and 6.7 × 10^−4^ mm/N with relatively low hysteresis of 4.4 × 10^−5^ mm/N and 1.9 × 10^−5^ mm/N, were measured, respectively [96]. However, for the meander configuration (third case), only a considerably low correlation between the applied force and the length change of the optical fiber was measured [96]. From the results obtained in [96], the authors have deduced that for periodic spatial variation of the optical fiber, and thus with less bonding length, the bonding between the optical fiber and the textile filament is weaker and, hence, the functionalized textile-based reinforcement structure is less sensitive to applied force [96]. Based on these experiments, a minimum length of 150 mm for the bonding length between the optical fiber and the textile filament was recommended [96].

### 4.4. Sensor Response of the Integrated FOSs

**Figure 17:**
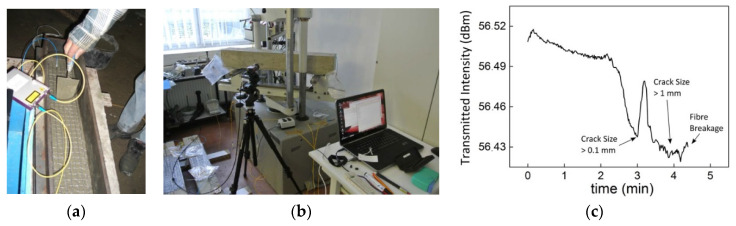
Embedding of the functionalized TNS into the concrete block (**a**), the experimental set-up (**b**), and the obtained results for the functionalized TNS to determine cracks of concrete structures (**c**) Reprinted with permission from Ref. [51]. Copyright 2016 Procedia Technology.

The response from the optical glass fiber inside the functionalized TRC that are embedded into concrete elements were investigated in [51,68] for TNS and in [97] for FCS. The TNS were functionalized with FOSs in order to design concrete crack sensors and, thus, to determine the early failure of structures [51,68]. For this task, the TNS was designed to transfer cracks of the concrete structure to the optical glass fiber. Moreover, the optical glass fiber inside the TNS was pre-strained in order to amplify crack transfer from the concrete structure and, thus, to break even at relatively small cracks. The sensor performance was evaluated by embedding the functionalized TNS into concrete blocks (100 × 15 × 15 cm^3^). This was done by breaking the concrete blocks at defined locations using a three-point bending test and measuring the crack size and the attenuation of the optical fiber inside the TNS. The embedding of the functionalized TNS into the concrete block, the experimental set-up, and the obtained results, are illustrated in Figure 17 [51]. While performing the experiments and before the breakage of the optical glass fiber, a light attenuation of the optical glass fiber of less than 0.09 dB was obtained [51]. Furthermore, the optical fiber inside the TNS broke at a crack size of 1.4 mm, indicating the failure of the structure [51].

**Figure 18:**
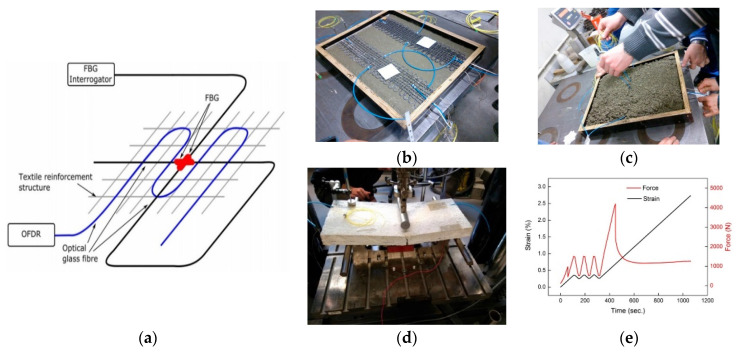
Experimental set-up to characterize the response of FOSs for point-based and distributed sensing (**a**), textile-based carbon reinforcement structures embedded in concrete blocks (**b**,**c**), the three-point-bending test (**d**), and the corresponding applied strain and force (**e**) [97].

FCS were investigated for point as well as for distributed-based fiber optic sensor configurations in [97]. For the point sensor configuration, two FBG sensors were used, which were arranged perpendicular to each other inside the functionalized textile-based reinforcement structure. For the distributed sensor configuration, the OFDR technique was applied and the optical fiber inside the functionalized TRC structure was arranged in a meander shape in order to analyze the spatial resolution, i.e., the spatial resolution of the FCS and, thus, of the sensor element itself. Both fiber optic sensor configurations are illustrated schematically in Figure 18 [97]. The dimensions of the fabricated functionalized textile-based carbon reinforcement structure were 500 × 110 mm^2^ and the structure was fabricated using 1600 tex carbon filaments. Once the fabrication of the functionalized textile-based carbon reinforcement structure was complete, the entire structure was embedded in concrete blocks and cured over a period of at least 28 days. No optical fiber broke during the embedding process and only an insignificant light attenuation was measured before and after the embedding of sensors into concrete, which was mainly attributed to connector tolerances. For the interrogation of the FBG sensors, a broadband light source, a 3 dB fused fiber coupler and a spectrometer were used. For the OFDR, the Luna ODiSI-B from Luna Innovations Incorporated was applied, with a spatial strain resolution of 1.28 mm. Strain was induced to the concrete block, i.e., with embedded functionalized textile-based reinforcement structure, using a three-point bending test.

**Figure 19:**
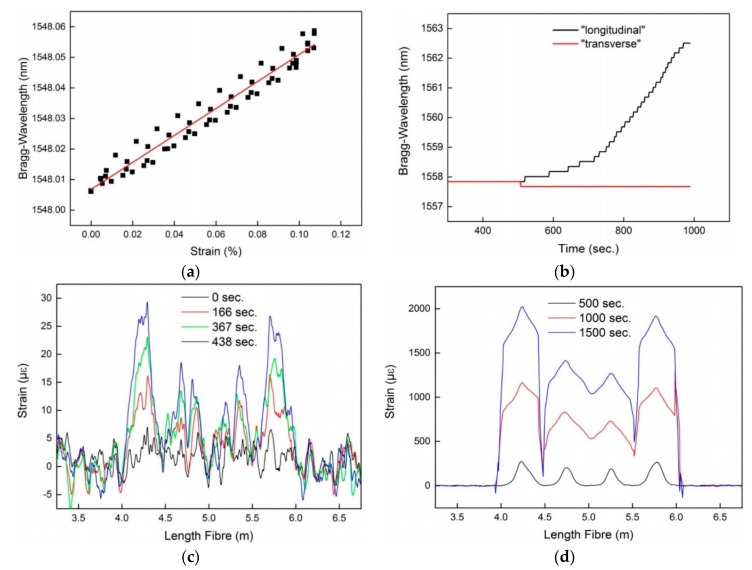
Obtained sensor response: Bragg-wavelength shift due to applied strain (**a**), discrimination between longitudinal and transverse applied strain (**b**), strain resolution of OFDR for low applied strains (**c**) and high applied strain (**d**) levels [97].

The responses of the FBG sensors and the OFDR are shown in Figure 19. From the FBG sensor response, the authors have deduced that depending on the position of FOSs relative to the load, the direction of the force between transverse and longitudinal directions, can be discriminated and the temperature cross-sensitivity of the FOS can be compensated [96]. Moreover, a linear response was obtained to an applied strain of 0.44 nm/% with a relatively low hysteresis (0.011%) for the FBG sensors [97]. In the case of the distributed FOS configuration and the applied OFDR technique, it was demonstrated in [97] that the spatial strain profile of the optical fiber of the functionalized textile-based reinforcement structure can be successfully monitored within concrete elements by measuring four load points while performing the three-point bending test (due to the meander shape, the optical fiber was exposed to the applied force four times). In addition, the authors observed a broadening of the measured strain peaks for large loads, which has a negative effect on the spatial resolution of the OFDR.

## 5. Summary

The recent growth in the wearable sensor technology market, especially in the field of biomedical and robotics sectors, indicates a clear direction that could be predicted for civil infrastructure of the future. It can be inferred that textile reinforcement will take over its conventional counterparts, and civil infrastructure will be fully embedded with sensors and other capabilities to ensure its safety, comfort, and long-term durability while minimizing the otherwise expected carbon footprint. In order to achieve these targets, reliable and cost-effective sensors need to be in place. Much like the concrete reinforcement sector, the infrastructure monitoring sensors sector has also seen a dramatic shift towards new methods of sensing, i.e., adopting fiber optic sensors as opposed to conventional SHM schemes such as strain gauges.

In terms of functionalizing these reinforcement structures with sensors for SHM applications, FOSs are the best candidates due to their advantages, such as their small size, light weight, and remote interrogation and multiplexing capability. Optical fibers can be integrated during the manufacturing process of the TRCs in large volumes or could be tailor-made. Thus, such structures can be fabricated economically and optimized for specific target applications. When FCSs are equipped with optical fibers, Bremer et al. demonstrated in [97] that the corresponding sensor response depends linearly on the applied strain with a relatively low hysteresis and, depending on the integration direction, the load direction can be discriminated between transverse and longitudinal loads. Moreover, in terms of distributed sensing, it could also be verified that integrated optical fibers can be applied to determine the applied strain profile. However, depending on the magnitude of the applied load, the obtained load peak tends to broaden, and this reduces the spatial resolution. Therefore, in [96] it was deduced that for the FOS response, the bonding length between the optical fiber and the textile-base reinforcement structure is important, i.e., that the bonding length is long enough and thus avoids directional variations of the optical fiber at the point of measurement, so that the applied load can be sufficiently transferred on to the FOS. Moreover, it was demonstrated that when embedding the functionalized textile-based carbon reinforcement structure into highly alkaline concrete, the applied PVA coating, which is relatively inert, provides substantial resistance against deterioration of the structure [96]. In addition, functionalized TNS designed for the reinforcement and crack detection of concrete structures have been reported in [51]. The optical glass fiber was pre-strained during the fabrication process of the functionalized TNS in order to enhance the crack detection mechanism. Furthermore, when embedding the functionalized TNS into concrete, an insignificant light attenuation of 0.09 dB and a relatively high sensitivity of crack detection, i.e., of ≥1.4 mm, was observed. The performance of functionalized TNS and FCS are summarized for comparison in Table 1. Future directions of functionalized TRCs clearly point towards increased exploitation of Rayleigh, Brillouin, and/or Raman scattering for fiber-optic sensor networks, their implementation for structural health monitoring, as well as the combination with artificial intelligence algorithms for signal analysis and sensitivity enhancement, see e.g., [98,99]. 

## Figures and Tables

**Table 1:** Comparison of functionalized TNS and FCS. Values obtained from [51,68,95,96].

**Host Structure**	**Textile Net Structures (TNS)**	**Functionalized Carbon Structure (FCS)**
Sensing mechanism	Point-based (e.g., power meter) andDistributed (OTDR)	Point-based (e.g., FBG, EFPI),Quasi-distributed (FBG network), andDistributed (e.g., DAS, OFDR, BOTDA)
Sensing parameter	Cracks	Strain
Sensitivity	≥1.4 mm	0.44 nm/%
Hysteresis	Not specified	0.011%
Spatial resolution	Not specified	≤0.5 m for distributed sensing
Fiber type	Single-mode optical glass fiber	Single-mode optical glass fiber
Optical attenuation	≤0.09 dB	Insignificant light attenuation due to connector tolerances
Fabrication technology	Reel-to-reel knitting technique	Modified embroidery technique
Estimated cost	Low	Moderate
Highlight	Pre-strained optical glass fiber	Relatively inert due to PVA coating
